# Overpromoted and underregulated: National binding legal measures related to commercially produced complementary foods in seven Southeast Asian countries are not fully aligned with available guidance

**DOI:** 10.1111/mcn.13588

**Published:** 2023-12-13

**Authors:** Jessica Blankenship, Tuan Nguyen, Jessica M. White, Jane Badham, Paul Zambrano, Duong Vu, Ha‐Anh Nguyen, Jennifer Cashin, Roland Kupka

**Affiliations:** ^1^ UNICEF East Asia Pacific Regional Office Bangkok Thailand; ^2^ Alive and Thrive East Asia Pacific, FHI 360/FHI Solutions Hanoi Vietnam; ^3^ JB Consultancy Johannesburg South Africa; ^4^ Viet Nam Food Administration Hanoi Vietnam

**Keywords:** Codex, commercially produced complementary foods, legislation, policies, standards

## Abstract

The market for commercially produced complementary foods (CPCF) is rapidly expanding in Southeast Asia; however, the existence and content of mandatory national policies, standards and legislation (binding legal measures) for CPCF in the region is unclear. To assess the status of national binding legal measures for CPCF in Southeast Asia, a legal and policy desk review was conducted in seven countries (Cambodia, Laos People's Democratic Republic, Indonesia, Malaysia, Philippines, Thailand and Viet Nam). The alignment of the national binding legal measures relevant to CPCF was assessed against guidance on CPCF nutrient composition and labelling requirements provided by Codex Alimentarius and the World Health Organization (WHO). Each of the seven countries had at least two national binding legal measures related to the nutrient composition or labelling of CPCF; however, there was limited alignment with the guidance from Codex and WHO. No country was fully aligned with the three CPCF‐specific Codex standards/guidelines and only one country was in full alignment with the recommendations related to the protection of breastfeeding from the ‘WHO Guidance on ending the inappropriate promotion of foods for infants and young children’. The findings of the review indicate that the existing national binding legal measures are insufficient to ensure that the CPCF sold as suitable for older infants and young children are nutritionally adequate and labelled in a responsible manner that does not mislead caregivers. Improved and enforced national binding legal measures for CPCF, in alignment with global guidance, are required to ensure that countries protect, promote and support optimal nutrition for children 6–36 months of age.

## Key messages

- Given the proliferation of commercially produced complementary foods (CPCF) in Southeast Asia, national binding legal measures are essential to ensure CPCF are nutritionally suitable for older infants and young children (IYC), and are labelled in a manner that does not mislead caregivers.
- All seven Southeast Asian countries included in this review were found to have at least two national binding legal measures related to the nutrient composition and/or labelling practices of CPCF. However, there was limited alignment between these binding legal measures and the available guidance documents.
- No country was fully aligned with the six relevant Codex Alimentarius (Codex) standards and only one country was fully aligned with guidance from the World Health Organization (WHO). Existing binding legal measures do not ensure that CPCF are nutritionally adequate and labelled in a responsible way that enables caregivers to make healthy choices for older IYC.
- Countries in Southeast Asia urgently require consolidated guidance on how to develop appropriate, legally binding and enforceable standards for CPCF nutrient composition and labelling. Existing guidance is fragmented and Codex standards are outdated and insufficient for this purpose.

## 1 INTRODUCTION

Adequate nutrition is a fundamental right for every child. Meeting the nutrient needs of older infants and young children 6–36 months of age (older IYC), however, can be challenging (Dewey, 2013) and the factors that influence how and what older IYC are fed are diverse and complex. In Southeast Asia, economic growth, increasingly global trade markets, rapid urbanization and an emergence of modern grocery retailers are contributing to a shift in consumption from traditional diets towards commercially processed foods that are often higher in salt, sugar and unhealthy fats, and lower in essential micronutrients (ASEAN, UNICEF and WFP, 2022; Development Initiatives, 2017; United Nations Department of Economic and Social Affairs, Populations Division, 2018). In Southeast Asia, commercially produced packaged foods marketed as suitable for older IYC—termed ‘commercially produced complementary foods’ (CPCF)—are often provided to children, because they are convenient (Schmied, 2020; UNICEF, 2019, 2021) and are promoted as healthy for older IYC (Walls, 2023). Well‐regulated CPCF can help to meet the micronutrient requirements of older IYC in a form that is familiar to caregivers, while addressing the increasing demand for convenience. Many CPCF in the market, however, are high in sugar, salt and/or use labelling practices that may mislead caregivers and should not be promoted for or provided to older IYC (Access to Nutrition Initiative, 2021; Bassetti et al., 2022; Sweet & Pereira, 2016). Strong binding legal measures, encompassing mandatory national policies, standards and legislation, are required to adequately regulate the nutrient composition and labelling practices of CPCF, to ensure they are nutritionally appropriate and promoted responsibly.

Guidance on the nutrient composition and labelling practices of CPCF is provided by Codex Alimentarius (Codex) and the World Health Organization (WHO). Countries are encouraged to incorporate the Codex standards/guidelines in full to their national legal measures as the minimum standard; however, adherence to Codex is voluntary and countries may modify any aspect of a standard or guideline to suit their context. Additionally, existing Codex standards/guidelines lack comprehensive nutrient composition standards for all CPCF product categories and exclude labelling requirements related to the protection of breastfeeding (Codex Alimentarious Commission, 1981, 2006, 2013). This gap was recognized in the ‘WHO Guidance on Ending the Inappropriate Promotion of Foods for Infants and Young Children’ through the recommendation that the relevant Codex standards for CPCF be updated and additional guidance be developed (WHO, 2016). In response, the WHO Regional Office for Europe developed a comprehensive set of nutrient composition and labelling recommendations based on the current global evidence base for older IYC nutrition (WHO, 2019, 2022). The WHO Europe nutrient and promotion profile model (WHO Europe NPM for CPCF) is the first guidance document to provide both nutrient composition and labelling requirements for all CPCF product categories.

The Consortium for Improving Complementary Foods in Southeast Asia (COMMIT) was established in 2021 to improve the diets of older IYC in Southeast Asia through stronger regulation of CPCF. COMMIT conducted a legal and policy desk review in seven Southeast Asia countries (Cambodia, Laos People's Democratic Republic [PDR], Indonesia, Malaysia, Philippines, Thailand and Viet Nam) to understand the status of existing national binding legal measures regulating CPCF in Southeast Asia and facilitate future policy action. The objective of the review was to assess the content of these legal measures relevant to CPCF nutrient composition and labelling, and to compare their alignment with recommendations provided through Codex and WHO guidance documents.

## 2 METHODS

### 2.1 Relevant guidance and CPCF product categories

There are three Codex standards/guidelines that specifically apply to CPCF are as follows: (1) standard for processed cereal‐based foods for IYC (Codex Alimentarious Commission, 2006); (2) standard for canned baby foods (Codex Alimentarius Commission, 1981) and (3) guidelines on formulated complementary foods for older IYC (Codex Alimentarius Commission, 2013). In addition, three other general Codex standards/guidelines are generally relevant to CPCF labelling as follows: (1) the general standard for the labelling of prepackaged foods (Codex Alimentarius Commission, 2018), (2) guidelines on nutrition labelling (Codex Alimentarius Commission, 2021) and (3) guidelines for use of nutrition and health claims (Codex Alimentarius Commission, 1997).

WHO provides guidance through two documents. First, guidance on what constitutes inappropriate promotion of CPCF is provided through ‘WHO's Guidance on Ending the Inappropriate Promotion of Foods for Infants and Young Children’ (referred to as the WHO Guidance) (WHO, 2016). Second, to address identified gaps in the provision of nutrient composition standards for all CPCF product categories and the WHO Regional Office for Europe developed a nutrient profile model (NPM) for CPCF (referred to as the WHO Europe NPM for CPCF) providing an extensive set of nutrient composition and labelling requirements for all CPCF product categories in Europe (WHO, 2019, 2022). The WHO Europe NPM for CPCF is based on recommendations from the WHO Guidance (WHO, 2016), the European Commission Directive 2006/141/EC (Official Journal of the European Union, 2013), the global expert group consensus for sugar (Buttriss, 2015; Mis et al., 2017; Vos, 2017) and sodium consumption (Fewtrell et al., 2017), and analysis of CPCF nutrient composition in the European Region (WHO, 2019). COMMIT adapted the WHO Europe NPM for CPCF to include minimum micronutrient requirements and incorporate additional detail on types of claims. This adapted model, referred to as the ‘adapted NPM for CPCF’, was utilized for the review.

The six Codex standards/guidelines of relevance to CPCF nutrient composition and labelling do not universally apply to all types of CPCF products. The three CPCF‐specific Codex standards/guidelines provide nutrient composition and labelling practice requirements that apply to specific categories of CPCF. The three general Codex standards/guidelines and the WHO Guidance provide labelling recommendations that apply to all types of CPCF but these documents do not provide nutrient composition recommendations. The WHO Europe NPM for CPCF is the only guidance document to provide both nutrient composition and labelling recommendations for all CPCF product categories and specifies five categories and 16 subcategories of CPCF products (Table 1). The scope of the six Codex standards/guidelines and the WHO Guidance is mapped against the WHO Europe NPM for CPCF product categories in Supporting Information: Table S1.

**Table 1:** CPCF product categories identified in the WHO Europe NPM for CPCF.

Categories	Contents
Category 1	Dry, powdered and instant cereal/starchy food
1.1	Dry or instant cereals/starch
Category 2	Soft–wet spoonable, ready‐to‐eat foods, typically smooth or semipuréed packaged in jars or pouches and can be spoon‐fed
2.1	Dairy‐based desserts and cereal products
2.2	Fruit purée with or without addition of vegetables, cereals or milk
2.3	Vegetable‐only purée
2.4	Puréed vegetables and cereals
2.5	Puréed meal with cheese (but not meat or fish) mentioned in the name
2.6	Puréed meal with meat or fish mentioned as first food in product name
2.7	Puréed meals with meat or fish (but not named first in product name)
2.8	Purées with only meat, fish or cheese in name
Category 3	Meals with chunky pieces, often sold in trays or pots for infants and young children
3.1	Meat, fish or cheese‐based meal with chunky pieces
3.2	Vegetable‐based meal with chunky pieces
Category 4	Dry finger foods and snacks
4.1	Confectionery, sweet spreads and fruit chews
4.2	Fruit (fresh or dry whole fruit or pieces)
4.3	Other snacks and finger foods
Category 5	Juices and other drinks
5.1	Single or mixed fruit juices, vegetable juices or other nonformula drinks
5.2	Cow's milk and milk alternatives with added sugar or sweetening agent

Abbreviations: CPCF, commercially produced complementary food; NPM, nutrient profile model; WHO, World Health Organization.

### 2.2 Identification of the binding legal measures

Key government and civil society stakeholders were identified in each of the seven countries and their assistance was requested in identifying binding legal measures relevant to CPCF. Relevant binding legal measures were defined as national policies, standards and legislation regulating the nutrient composition and/or labelling requirements specific to CPCF or applicable to all foods, including CPCF. Twenty‐two stakeholders from the seven countries completed a survey on Google Forms between August 27 and September 6, 2021. In addition, a regional law firm (DFDL Legal, Tax and Investment Expertise in Southeast Asia) was contracted to assist with policy identification in Indonesia, Malaysia, Philippines and Thailand from December 2021 to March 2022. Documents only available in the national language of the seven countries were translated into English using Google Translate for text and Google Lens for images and then reviewed for content by native language researchers. A total of 87 national binding legal documents were identified. The research team reviewed each document and selected 35 for further analysis; 15 specific documents to CPCF and 20 generally relevant documents to CPCF. The 52 excluded documents did not relate to CPCF specifically or generally. The complete list of analysed national binding legal documents is provided in Supporting Information: Table S2.

### 2.3 Development of data extraction form

A Legal Measures Analysis Checklist was created to extract relevant information from each of the 35 documents selected for analysis (Supporting Information: Table S3). The checklist incorporated content related to CPCF nutrient composition and labelling from the six Codex standards/guidelines, the WHO Guidance and the adapted NPM for CPCF. A comparison of the content of Codex standards/guidelines and WHO Guidance with the adapted NPM for CPCF is provided in Supporting Informations: Tables S4 and S5.

### 2.4 Extracting information from identified national binding legal measures

Two legal experts reviewed all documents selected for analysis and applied the Legal Measures Analysis Checklist to compare their content to the nutrient composition and labelling recommendations in (1) the six Codex standards/guidelines, (2) the WHO Guidance and (3) the adapted NPM for CPCF. Each legal expert reviewed the selected national binding legal measures independently. Individual responses were then jointly reviewed by both experts to finalize the framework for each country. Final responses were validated by co‐authors and government officials familiar with the binding legal measures in each of the seven countries.

For each component of the Legal Measures Analysis Checklist, a broad three‐category classification was applied to assess the existence of national binding legal measures and their alignment with the CPCF nutrient composition and labelling recommendations in Codex, the WHO Guidance and the adapted NPM for CPCF: no binding legal measures; partial alignment; or full alignment. ‘No binding legal measures’ indicates that none of the identified measures refer to any of the recommended CPCF nutrient composition or labelling practices. ‘Partial alignment’ indicates that the measures include some component of the recommended CPCF nutrient composition and labelling practices, but not all. ‘Full alignment’ indicates that the measures include all components of the recommended CPCF nutrient composition and labelling practices.

## 3 RESULTS

All seven countries included in the review had at least two binding legal measures generally relevant to the nutrient composition and/or labelling of CPCF. The number of binding legal measures identified were two (Cambodia and Malaysia), four (Thailand), five (Lao PDR and Viet Nam), eight (Indonesia) and nine (Philippines). All seven countries had at least one CPCF‐specific binding legal measure, ranging from one in Cambodia, Malaysia and Lao PDR to three in the Philippines and Viet Nam. The dates when the binding legal measures became effective ranged from 1986 in the Philippines to 2021 in Indonesia, Malaysia and the Philippines.

The alignment of the binding legal measures with the CPCF nutrient composition and labelling recommendations outlined in the six Codex standards/guidelines and the WHO Guidance varied by country (Table 2). Two countries (Malaysia and Viet Nam) were in full alignment with at least one of the three CPCF‐specific Codex standards/guidelines. Cambodia, Indonesia, Malaysia and the Philippines were each in full alignment with at least one of the three general Codex standard/guidelines. Lao PDR was the only country in full alignment with the WHO Guidance recommendations specific to breastfeeding messaging on CPCF. Thailand was not in full alignment with any of the six Codex standards/guidelines or the WHO Guidance.

**Table 2:** Alignment of national binding legal measures with Codex standards/guidelines and WHO guidance.

	Cambodia	Indonesia	Lao PDR	Malaysia	Philippines	Thailand	Viet Nam
Codex standards/guidelines specific to CPCF							
Codex standard for processed cereal‐based foods for IYC (CXS 74‐1981)							
Codex standard for canned baby foods (CXS 73‐1981)							
Codex guidelines on formulated complementary foods for older IYC (CAC/GL 8‐1991)							
General Codex standards/guidelines generally relevant to CPCF							
Codex general standard for the labelling of pre‐packaged foods (CXS 1‐ 1985)							
Codex guidelines on nutrition labelling (CXG 2 – 1985)							
Codex guidelines for use of nutrition and health claims (CAC/GL 23 – 1997)							
WHO Guidance							
WHO Guidance on ending the inappropriate promotion of foods for IYC (2016)							

*Note*: 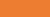, no binding legal measures; 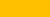, partial alignment; 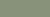, full alignment.

Abbreviations: CPCF, commercially produced complementary food; IYC, infants and young children; PDR, People's Democratic Republic; WHO, World Health Organization.

Table 3 compares national binding legal measures to the adapted NPM for CPCF nutrient composition requirements. Where relevant, the degree of alignment is noted for specific CFCF product categories. Binding legal measures in Indonesia, Malaysia and Viet Nam were fully aligned with the adapted NPM for CPCF on total fat and protein requirements for specific food categories and partially aligned on added sugar, sodium, fruit content and total sugar content requirements for specific food categories. Indonesia's binding legal measures contained the most comprehensive nutrient composition standards with eight of the 12 requirements included and binding legal measures covering each of the 16 CPCF product categories. Cambodia and Lao PDR had no binding legal measures regulating the nutrient composition of CPCF. The Philippines had one measure partially aligned to micronutrient content requirements of CPCF and Thailand had two measures partially aligned to requirements for no added sugar and the sodium threshold (Table 3).

**Table 3:** Comparison of national binding legal measures relating to CPCF nutrient composition and adapted NPM for CPCF requirements by CPCF product category.

Requirements	Relevant CPCF categories	Cambodia	Indonesia	Lao PDR	Malaysia	Philippines	Thailand	Viet Nam
Nutrient composition requirements								
No added sugars or sweetener	All				Categories 1, 4			Categories 1, 4
					Categories 2, 3			Categories 2, 3
Food category‐specific sodium limits	All							Categories 1, 4
								Categories 2, 3
Food category‐specific total fat limit	All		Categories 2, 3, 4		Categories 1, 4			Categories 1, 4
			Category 1		Categories 2, 3			Categories 2, 3
Food category‐specific limit on percentage of fruit content	Categories 1, 2 and 3		Category 1		Category 1			Category 1
			Categories 2, 3		Categories 2, 3			Categories 2, 3
Food category‐specific total sugar limit	Category 4.3							
Food category‐specific protein limit	Category 1 and some category 2 and 3		Category 1		Category 1			Category 1
			Categories 2, 3		Categories 2, 3			Categories 2, 3
Food category‐specific minimum for energy density	Category 2							
Additional recommendations in the adapted NPM for CPCF that are not requirements								
Front‐of‐pack high sugar warning^a^	Categories 1, 2 and 3				Category 1^b^			Category 1^b^
					Categories 2, 3			Categories 2, 3
National standards for micronutrient content of CPCF	All categories							

*Note*: 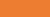, no binding legal measures; 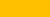, partial alignment; 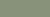, full alignment.

Abbreviations: CPCF, commercially produced complementary food; NPM, nutrient profile model; PDR, People's Democratic Republic.

^a^ Required if the percentage of energy from total sugar content exceeds category‐specific thresholds.

^b^ Malaysia and Viet Nam binding legal measures include a maximum total sugar requirement for sale of a CPCF. Neither country has a front of pack high sugar warning requirement.

National binding legal measures in each of the seven countries were at least partially aligned with labelling recommendations in the adapted NPM for CPCF (Table 4). Five countries (Indonesia, Laos PDR, Malaysia, Thailand and Viet Nam) were in full alignment with the requirement to provide the percentage by weight of fruit, water and protein in the ingredients list. Lao PDR and Malaysia were fully aligned with several requirements to promote and protect breastfeeding. None of the seven countries were in full alignment with the requirements of (1) no compositional, nutritional, health, marketing or other claims; (2) minimum age restriction of 6 months of a maximum age restriction of 12 months for specific food categories; (3) product name reflects ingredients in descending order as per ingredients list; and (4) instructions not to consume soft foods via pack spout.

**Table 4:** Comparison of national binding legal measures relating to labelling practice requirements in seven COMMIT countries in Southeast Asia with the adapted NPM for CPCF.

Requirements	Relevant CPCF categories	Cambodia	Indonesia	Laos PDR	Malaysia	Philippines	Thailand	Viet Nam
No compositional, nutritional, health, marketing, or other claims								
No compositional, nutritional, health, marketing or other claims permitted on packs or labels	All categories							
Promotion and protection of breastfeeding								
All products must state the suitable age of introduction with a minimum age of at least 6 months written on the label. All Category 2 products must additionally state a maximum age of 12 months written on the label.	All categories							
No product should include any image, text or other representation that might suggest suitability for infants under the age of 6 months	All categories				Categories 1, 4			
					Categories 2, 3			
All products must include a statement on the important of continued breastfeeding for up to 2 years or beyond	All categories				Categories 1, 4			
					Categories 2, 3			
No product should include any image, text or other representation that is likely to undermine or discourage breastfeeding, or that makes a comparison with breastmilk or that suggests that the product is nearly equivalent or superior to breastmilk.	All categories							
No product should include any image, text or other representation that might recommend or promote bottle feeding.	All categories							
Product name clarity								
The front of pack product name and legal product name must list ingredients in order as per ingredient list to reflect decreasing proportional content	All categories							
Ingredient list clarity								
If the product contains fruit, the ingredient list must clearly indicate the proportion (%) by weight of fruit (fresh or powdered/processed	Categories 1, 2, 3 and 4.3							
If the product contains added water, the ingredient list must clearly indicate the proportion (%) by weight of water	Categories 2 and 3							
If the product contains a traditional source of protein, the ingredient list must clearly indicate the proportion (%) by weight of protein.	Categories 2.6, 2.7, 2.8 and 3.1							
Instructions not to consume soft foods via pack spout								
Ready‐to‐eat pureed foods sold in packs with a spout must include a clear statement to discourage caregivers from allowing IYC to suck the food directly via the spout and a statement that warns against choking hazard.	Category 2 products with a spout							

*Note*: 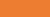, no binding legal measures; 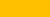, partial alignment; 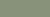, full alignment.

Abbreviations: COMMIT, Consortium for Improving Complementary Foods in Southeast Asia; CPCF, commercially produced complementary food; NPM, nutrient profile model; IYC, infants and young children.

Table 5 compares national binding legal measures to the adapted NPM for CPCF requirement prohibiting the promotion of three CPCF product categories: Category 4.1: sweet confectionary, sweet spreads and fruit chews; Category 5.1: fruit, vegetable juices and non‐breastmilk substitute (BMS) beverages; and Category 5.2: cow's milk and milk alternatives with added sugar or sweetening agent. There were no binding legal measures restricting the promotion of confectionary and non‐BMS beverages in any of the seven countries.

**Table 5:** Comparison of national binding legal measures relating to adapted NPM for CPCF requirement to prohibit the promotion of specific CPCF product categories.

Requirements	Relevant CPCF categories	Cambodia	Indonesia	Lao PDR	Malaysia	Philippines	Thailand	Viet Nam
CPCF products prohibited from promotion								
Sweet confectionery, sweet spreads and fruit chews	Category 4.1							
Single or mixed fruit juices, vegetable juices, or other non‐BMS drinks	Category 5.1							
Cow's milk and milk alternatives, with added sugar or sweetening agent	Category 5.2							

*Note*: 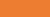, no binding legal measures; 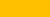, partial alignment; 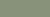, full alignment.

Abbreviations: BMS, breastmilk substitute; CPCF, commercially produced complementary food; NPM, nutrient profile model; PDR, People's Democratic Republic.

## 4 DISCUSSION

All seven Southeast Asian countries included in this review were found to have at least two national binding legal measures related to the nutrient composition and/or labelling of CPCF. However, there was limited alignment between these binding legal measures and available guidance documents from WHO and Codex. There was a higher occurrence of partial or full alignment with the six Codex standards/guidelines relevant to CPCF than with the WHO Guidance or adapted NPM for CPCF. This finding can be partially attributed to the timing of guidance availability; relevant Codex standards/guidelines for CPCF were first published several years ago (the earliest being in 1981), whereas the WHO Guidance and WHO Europe NPM for CPCF published in 2016 and 2022, respectively, providing limited time for countries to incorporate their recommendations into binding legal measures. Additionally, alignment with Codex is a national priority as Codex is frequently referenced in World Trade Organization agreements and disputes (Russ et al., 2021), making alignment important not only for public health policy but for international trade.

Codex recommendations for nutrient composition of CPCF are guided by three Codex standards/guidelines (CSX 074‐1981, CXS 73‐1981 and CAC/GL 8‐1991). However, except for Indonesia, Malaysia and Viet Nam, countries included in this analysis had no or few regulations relating to CPCF nutrient composition. This absence may be attributed to the lack of nutrient composition guidance in the Code of Marketing of Breast‐milk Substitutes (the Code), which is a priority for infant and young child feeding policies in low and middle‐income countries. Recently, the publication of the WHO Guidance with its call to develop national NPM's for CPCF, in conjunction with the growing CPCF market, has created both interest and urgency within countries to develop robust CPCF nutrient composition and labelling standards at the national level (WHO, 2016). The existing Codex standards/guidelines are insufficient, however, to meet the demand for comprehensive guidance on CPCF regulation from countries. Although the number and categories of CPCF have proliferated rapidly in recent years (Euromonitor International, 2022), comprehensive nutrient composition requirements in Codex exist only for cereal based CPCF food categories. The Codex standard for canned baby food, covering all pureed and ready‐to‐eat CPCF meal food categories, only provides nutrient composition standards for sodium. The Codex guidelines on formulated complementary food provides recommendations for energy density, percentage of energy derived from fat and protein for CPCF on a dry weight basis and reference for fortification level of vitamins and minerals; however, this document excludes CPCF food categories covered by the Codex standard for processed cereal based foods for infants and children and Codex standard for canned baby foods, significantly limiting the scope of the guidelines (Codex Alimentarius Commission, 2013). WHO called for the updating of the existing Codex standards/guidelines specific to CPCF (WHO, 2016) but possibly noting the lengthy and complex Codex process, promoted the national adaptation and adoption of additional guidance documents, such as the WHO Europe NPM for CPCF. The WHO Europe NPM for CPCF is the first guidance document to provide comprehensive nutrient composition recommendations for all categories of CPCF, making it a robust resource for countries inside and outside of Europe.

Recommendations on labelling practices for CPCF are provided by all six Codex standards/guidelines, WHO Guidance and the WHO Europe NPM for CPCF. However, alignment between national binding legal measures and global guidance varied substantially by country and labelling requirement category. Greatest alignment with the adapted NPM for CPCF was found where the requirement did not deviate from the Codex standard/guideline it was based on. For example, the binding legal measures for all countries were aligned with requirements for ingredient list clarity whose requirements are the same between the adapted NPM for CPCF and Codex general standard for labelling of prepackaged foods. The requirement for product name clarity, without a Codex equivalent, was not in full alignment with any binding legal measures. The requirement for no nutrition or health claims was noteworthy as all countries permitted the use of some form of these claims for older IYC despite Codex explicitly not permitting their use, except where specifically provided for in national legislation. Indonesia was the only country to prohibit the use of any nutrition or health claims with this restriction applied to all food products intended for infants under 12 months of age.

The lack of alignment with the Codex guidelines for the use of nutrition and health claims underscores the role of commercial influence in the development of national binding legal measures, well‐documented globally and in Southeast Asia (Baker, Russ, et al., 2021; Pettigrew et al., 2022). The infant and young child food industry uses lobbying and political financing to both directly and indirectly influence the regulatory scope and scale of national binding legal measures for CPCF. These strategies are well‐documented in the Philippines, where the private sector has continually applied pressure to weaken the national Milk Code regulating breastmilk substitutes and CPCF (Baker, Zambrano, et al., 2021). Despite having a national Code law in place, the Philippines lacked full alignment with the WHO Guidance recommendations for the protection and promotion of breastfeeding. Of the six countries with a mandatory Code law in place, Laos PDR alone fully aligned all the requirements for the promotion and protection of breastfeeding. Lao PDR recently passed national legislation on the Code through the ‘Decree on Food Products and Feeding Equipment for Infants and Toddlers’ in 2019 (Government of Lao PDR, 2019) and was thus able to include recommendations from the WHO Guidance. Notably, Malaysia was in full alignment with two of the five recommendations related to the promotion and protection of breastfeeding, despite not having a mandatory Code law, providing an example of how articles of the Code can be integrated into existing national binding legal measures.

The WHO Europe NPM for CPCF recommends prohibiting the promotion of CPCF classified as confectionary or beverages. Both CPCF confectionary and beverages provide little beneficial nutrition aside from energy from sugar and are often very sweet, posing risks to dental health and contributing to a preference for sweet tastes in older IYC (WHO, 2015, 2019). The concept of prohibiting promotion of food products is relatively new and is well recognized as being complex, likely to be contested by the food industry (Garton et al., 2021; Sing et al., 2022) and requiring strong capacity for monitoring and enforcement by governments. Countries, however, are increasingly passing restrictions on the promotion of food and drinks high in fat, sugar and salt (Correa et al., 2020; Lee et al., 2017; Taillie et al., 2019; Watt et al., 2020). These restrictions involve the inclusion of front of pack warning labels, limits on advertising through television, online media, radio and in public spaces such as public transportation and billboards. There is precedence for the prohibition of specific foods from promotion as being suitable for older IYC in Southeast Asia. In 2018, Indonesia's Food and Drug Supervisory Agency (Badan Pengawas Obat dan Makanan [BPOM]) prohibited the promotion of sweetened condensed milk for children under the age of 5 years with restrictions on the product labelling and advertisement on children's television programmes (BPOM, 2018). Malaysia mandates the inclusion of the statement ‘Not suitable for infants’ on all containers of condensed and evaporated milk and additionally prohibits the use of images of infants on the labels of products containing milk or milk products (Laws of Malaysia, 1985). They further prohibit the sale of jelly confectionary containing konjac and require the statement ‘Not suitable for children below 3 years’ on all jelly confectionaries.

A clear finding from this assessment is the lack of a single guidance document providing nutrient composition and labelling requirements for all categories in CPCF. The WHO Europe NPM for CPCF provides extensive requirements for both nutrient composition and labelling; however, as it is intended to be used as tool to measure and compare quality of CPCF, it does not include production standards provided by Codex standards/guidelines. The available guidance documents together form a comprehensive manual to countries in the revision and development of national CPCF standards. However, consolidation of the guidance documents is required to provide countries with comprehensive and clear guidance to develop national binding legal measures for CPCF. To this end, the COMMIT Initiative developed a compendium of all nutrient composition, labelling practice and production requirements across the Codex standards/guidelines, the WHO Guidance and the WHO Europe NPM for CPCF (Blankenship, 2023).

To the best of our knowledge, this is the only review conducted of CPCF national binding legal measures to assess alignment with available CPCF guidance. WHO's global nutrition policy review 2016–2017 identified the presence of national binding legal measures relevant to maternal, infant and young child nutrition, including the presence of regulation on marketing of complementary foods (WHO, 2018). However, the content of these national binding legal measures was not further explored nor compared against available guidance. A review of national binding legal measures pertaining to CPCF in Cambodia, Nepal, Senegal and Tanzania was conducted in 2016 (Sweet & Pereira, 2016), summarizing relevant content for labelling practice requirements; however, this work was conducted before the publication of the WHO Guidance and the comprehensive WHO Europe NPM for CPCF and therefore did not directly review content against these guidance documents. The WHO Europe NPM for CPCF has been, to date, used to benchmark CPCF products purchased in Europe (WHO, 2019) and in Southeast Asia (Access to Nutrition Initiative, 2021; Bassetti et al., 2022). Although benchmarking assessments are a vital component of understanding the quality of CPCF for sale globally, a detailed review of the content of national binding legal measures pertaining to CPCF provides essential documentation of the scope and depth of existing measures and together with the benchmarking assessment findings, clarifies priorities for strengthening existing measures. This assessment is limited to the available guidance on CPCF nutrient composition and labelling. However, the available guidance from Codex is recognized in some instances as being outdated and insufficient to regulate the nutrient composition of all categories of CPCF (WHO, 2016). Codex standards/guidelines should be considered a minimum standard for production rather than a global standard as they are highly influenced by the food industry and often represent a compromise between health and nutrition experts and industry lobbyist (Silva et al., 2022). To address the limitations of Codex standards/guidelines, this review additionally compared all national binding legal measures to the adapted NPM for CPCF, comprised recently updated comprehensive nutrient composition and labelling practice standards based on the current global evidence base for older IYC nutrition and adapted for the Southeast Asia context through the inclusion of micronutrient content requirements.

## 5 CONCLUSION

The availability and variety of CPCF marketed as suitable for older IYC in Southeast Asia is increasing. Although each of the seven countries included in this review had at least two binding legal measures related to the nutrient composition and/or labelling of CPCF, there was limited alignment between these national binding legal measures and available global guidance. As a result, many CPCF products promoted in Southeast Asia as suitable for older IYC are neither nutritionally adequate nor labelled in a responsible manner that does not mislead caregivers. Improved, comprehensive and enforceable national binding legal measures for CPCF are required to ensure that countries protect, promote and support optimal nutrition for older IYC.

## AUTHOR CONTRIBUTIONS

Jessica Blankenship and Roland Kupka are UNICEF staff members, and Jessica M. White is a UNICEF consultant. The opinions and statements in this article are those of the authors and may not reflect official UNICEF policies.

## CONFLICT OF INTEREST STATEMENT

The authors declare no conflict of interest.

## Supporting information

Supporting Information.

Supporting Information.

Supporting Information.

Supporting Information.

Supporting Information.

## Data Availability

The data that support the findings of this study are available from the corresponding author upon reasonable request.
