# Supplementary material for: Overpromoted and underregulated: National binding legal measures related to commercially produced complementary foods in seven Southeast Asian countries are not fully aligned with available guidance
Source: Matern Child Nutr. 2023 Dec 13;19(Suppl 2):e13588. doi: 10.1111/mcn.13588 (PMC10719056; doi:10.1111/mcn.13588)
Supplement: Supplementary file 1 — Supporting Information. [file MCN-19-e13588-s004.docx]

**Supplemental Table 1** Classification of CPCF product categories included in the WHO Europe NPM for CPM by relevant Codex standards/guidelines and the WHO Guidance

| **WHO Europe NPM for CPCF product category** | **Codex Alimentarius Standards and Guidelines** | | | | | | **WHO Guidance on ending the inappropriate promotion of foods for infants and young children** |
| --- | --- | --- | --- | --- | --- | --- | --- |
|  | Standard for processed cereal-based foods for infants and young children  (CXS 74-1981) | Standard for canned baby foods  (CXS 73-1981) | Guidelines on formulated complementary foods for older infants and young children  **(**CAC/GL 8 –1991) | General standard for labelling of pre-packaged foods  (CXS 1 –1985) | Guidelines on nutrition labelling  (CXG 2 –1985) | Guidelines for use of nutrition and health claims  (CCA/GL 23-1997) |  |
| Category 1 |  |  |  |  |  |  |  |
| 1.1 Dry or instant cereals/starch | X |  |  | X | X | X | X |
| Category 2 |  |  |  |  |  |  |  |
| 2.1 Dairy-based desserts and cereal products |  | X |  | X | X | X | X |
| 2.2 Fruit purée with or without addition of vegetables, cereals, or milk |  | X |  | X | X | X | X |
| 2.3 Vegetable only purée |  | X |  | X | X | X | X |
| 2.4 Puréed vegetables and cereals |  | X |  | X | X | X | X |
| 2.5 Puréed meal with cheese (but not meat or fish) mentioned in the name |  | X |  | X | X | X | X |
| 2.6 Puréed meal with meat or fish mentioned as first food in product name |  | X |  | X | X | X | X |
| 2.7 Puréed meals with meat or fish (but not named as the first food in product name) |  | X |  | X | X | X | X |
| 2.8 Purées with only meat, fish, or cheese in name of product |  | X |  | X | X | X | X |
| Category 3 |  |  |  |  |  |  |  |
| 3.1 Meat, fish, or cheese-based meal with chunky pieces |  | X |  | X | X | X | X |
| 3.2 Vegetable-based meal with chunky pieces |  | X |  | X | X | X | X |
| Category 4 |  |  |  |  |  |  |  |
| 4.1 Confectionery, sweet spreads and fruit chews |  |  | X | X | X | X | X |
| 4.2 Fruit (fresh or dry whole fruit or pieces) |  |  | X | X | X | X | X |
| 4.3 Other snacks and finger foods | X |  |  | X | X | X | X |
| Category 5 |  |  |  |  |  |  |  |
| 5.1 Single or mixed fruit juices, vegetable juices, or other non-formula drinks |  |  | X | X | X | X | X |
| 5.2 Cow’s milk and milk alternatives with added sugar or sweetening agent |  |  | X | X | X | X | X |
