# Supplementary material for: Overpromoted and underregulated: National binding legal measures related to commercially produced complementary foods in seven Southeast Asian countries are not fully aligned with available guidance
Source: Matern Child Nutr. 2023 Dec 13;19(Suppl 2):e13588. doi: 10.1111/mcn.13588 (PMC10719056; doi:10.1111/mcn.13588)
Supplement: Supplementary file 2 — Supporting Information. [file MCN-19-e13588-s002.docx]

**Supplemental Table 2** Active binding legal measures for commercially produced complementary foods (CPCF)

|  | **Effective year** | **Type** | **Relevance to CPCF** | **Topics** |
| --- | --- | --- | --- | --- |
| **Cambodia** |  |  |  |  |
| 1. Prakas No.1045/2000 on Cambodian Standard CS 001-2000 Labelling of Food Products | 2000 | Mandatory | General | Defines general standards for all pre-packed foods. |
| 2. Sub-Decree No.133/2005 on Marketing of Products for Infant and Young Child Feeding | 2005 | Mandatory | Specific | Regulation about marketing CPCF and breastmilk-substitute. |
| **Indonesia** |  |  |  |  |
| 1. BPOM Regulation 24/2020 on Supervision of Processed Foods for Special Nutritional Needs | 2002 | Mandatory | Specific | All CPCF for children 6-24 months of age |
| 2. BPOM Regulation Number 13/2016 on Control of Claims on Processed Food Labels and Advertising | 2016 | Mandatory | General | All processed foods including CPCF 6-36 months of age |
| 3. BPOM Regulation No. 11/2019 | 2019 | Mandatory | General | All processed foods including CPCF 6-36 months of age |
| 4. BPOM Regulation No. 9/2016 | 2016 | Mandatory | General | All processed foods including CPCF 6-36 months of age |
| 5. Government Regulation 69/1999 | 1999 | Mandatory | General | All processed foods including CPCF 6-36 months of age |
| 6. BPOM Reg 20/2021 on Processed Food Labels | 2021 | Mandatory | General | All processed foods including CPCF 6-36 months of age |
| 7. BPOM Regulation 31/2018 | 2018 | Mandatory | General | All processed foods including CPCF 6-36 months of age |
| 8. Guidelines for the Implementation of Regulation of Complementary Foods for Breastfeeding (MP-ASI) for Micro, Small and Medium Enterprises | 2021 | Voluntary | Specific | All CPCF for children 6-24 months of age |
| **Laos** |  |  |  |  |
| 1. Law on Food No. 172/PS/2013 | 2013 | Mandatory | General | General rules about foods, food safety principles, food safety control, etc. |
| 2. Regulation on Labelling of Prepacked Food No.519/MoH/2009 | 2009 | Mandatory | General | General rules about labelling for any type of prepacked food. |
| 3. Decree on Food Products and Feeding Equipment for Infants and Toddlers No.472/GOL/2019 | 2019 | Mandatory | Specific | Regulation on the food products, feeding tools, BMS for infants and young children, management of these products, labelling and prohibitions. |
| 4. Implementation Guideline of the Decree on Food Products and Feeding Equipment for Infants and Toddlers No.1540/MoH/2020 | 2020 | Mandatory | General | General provisions and guidelines for the implementation of Decree No. 472/GOL/2019 |
| 5. Notification of Steering Committee for Food and Drug Administration No. 417/OCP/1997 - Appointment of National Codex Committee of Lao. | 1997 | Mandatory | General | Establishing National Codex Committee of Laos and defining its objectives and roles. |
| **Malaysia** |  |  |  |  |
| 1. Food Act (Laws of Malaysia Act 281) | 1983 | Mandatory | General | All foods |
| 2. Laws of Malaysia PU(A) 437/1985 Food Regulations with Amendments - Food Regulations | 1985 | Mandatory | Specific | All foods with Articles 390 and 391 specific to canned CPCF and cereal based CPCF |
| **Philippines** |  |  |  |  |
| 1. Executive Order No. 51 or the National Code of Marketing of Breastmilk Substitutes, Breastmilk Supplement and Other Related Products (“Milk Code”) | 1986 | Mandatory | Specific | Adopting a national Code of Marketing of BMS, Breastmilk Supplements and Complementary Foods |
| 2. Department of Health (DoH) circular No. 24 of 03/04/1987 | 1987 | Mandatory | Specific | Implementing rules of the Milk Code. |
| 3. Rules of Inter-Agency Committee created under the Milk Code – 26/05/1987 | 1987 | Mandatory | Specific | Establishes the rules of functioning of the Inter-Agency Committee to govern the advertising, promotion, and marketing of products within the scope the Milk Code. |
| 4. Bureau of Food and Drugs (BFAD) Administrative Order No. 4-A s. 1995 Guidelines on Micronutrient Fortification of Processed Foods | 1995 | Mandatory | General | Guidelines to improve nutritional quality to avoid over or under fortification that may create imbalance in the diet. |
| 5. Republic Act No.8976/2000 Establishing the Philippine Food Fortification Program and for Other Purposes | 2000 | Mandatory | General | Establishing Food Fortification Program in the Philippines |
| 6. DoH Administrative Order No.2006-0012 Revised Implementing Rules and Regulations of Executive Order No.51 - Milk Code | 2006 | Mandatory | Specific | Revises and updates DoH circular No. 24 of 03/04/1987 regarding the Implementing rules of the Milk Code. |
| 7. DoH Department Circular No. 2008- 0006 to All Milk Companies and Others Concerned | 2008 | Mandatory | Specific | Guidelines for the Labeling of breastmilk substitutes, infant formula, other milk products, foods and beverages, and other related products within the scope of the "Milk Code” |
| 8. DoH Administrative Order No.2014/0030 | 2014 | Mandatory | General | General rules about the labelling of prepackaged foods (including CPCFs) |
| 9. DOH Administrative Order No. 2021-0039 | 2021 | Mandatory | General | Establishes the national policy on the elimination of industrially produced trans-fatty acids for the prevention and control of non-communicable diseases. |
| **Thailand** |  |  |  |  |
| 1. Notification of the Ministry of Public Health No.158/1994 Re: Supplementary Food for Infants and Young Children | 1994 | Mandatory | Specific | Rules about CPCF production. |
| 2. Notification of the Ministry of Public Health No.367/2014 Re: Labelling of Pre-packaged Foods | 2014 | Mandatory | General | General rules about labelling prepacked foods. |
| 3. Notification of the Ministry of Public Health No.383/2017 Re: Labelling of Pre-packaged Foods (No.2) | 2017 | Mandatory | General | General rules about labelling prepacked foods. |
| 4. Control of Marketing Promotion of Infant and Young Child Food Act, 2017. | 2017 | Mandatory | Specific | Regulation for marketing CPCF and breastmilk-substitute. |
| **Viet Nam** |  |  |  |  |
| 1. Circular No. 23/2012/TT-BYT of November 15, 2012, Issuing National Technical Regulations on Processed Cereal-based Foods for Infants from 6th Month on and Young Children up to 36 Months of Age | 2012 | Mandatory | Specific | Standard for cereal based CPCF |
| 2. Circular No. 43/2014/TT-BYT Regulating the management of Functional Foods | 2014 | Mandatory | General | Standard for the use of fortification in all foods including CPCF |
| 3. Decree No.100/2014/ND-CP on the Trading and Use of Nutritious Products for infants, Feeding Bottles and Teats | 2014 | Mandatory | Specific | Regulation for marketing CPCF and breastmilk-substitute. |
| 4. Decree No.43/2017/ND-CP About Goods Labels | 2017 | Mandatory | General | General rules about labelling prepacked foods. |
| 5. TCVN 12441:2018 Guidelines for Formulated Supplementary Foods for Older Infants and Young Children | 2018 | Voluntary | Specific | Guidelines for non-cereal and non-canned based CPCF. |
