# Supplementary material for: Overpromoted and underregulated: National binding legal measures related to commercially produced complementary foods in seven Southeast Asian countries are not fully aligned with available guidance
Source: Matern Child Nutr. 2023 Dec 13;19(Suppl 2):e13588. doi: 10.1111/mcn.13588 (PMC10719056; doi:10.1111/mcn.13588)
Supplement: Supplementary file 3 — Supporting Information. [file MCN-19-e13588-s005.docx]

**Supplementary Table 3** Legal Measures Analysis Checklist

| Legal Measures Analysis Checklist | | | |
| --- | --- | --- | --- |
| Overview | 1 | Does the country have legally binding standards for commercially produced foods intended for children 6 to 36 months of age? | |
|  |  | Yes | No |
|  |  | Continue with question 2 | Skip to Question X |
|  |  |  |  |
|  | X | Does the country follow Codex standards and guidance for commercially produced foods intended for children 6 to 36 months of age? (Include reference to the specific Codex standards and guidance). | |
|  |  |  |  |
| Nutrient Content and Composition | 2 | Are there binding legal measures for the essential composition of commercially produced foods intended for children 6 to 36 months of age? (Essential composition includes specific definitions for food types, categories of foods intended for children under 3 and identified thresholds for the amount added) | |
|  |  | Yes | No |
|  |  |  |  |
|  | 3 | If yes, please include the specific binding legal measure for the following components: | |
|  |  |  | Suitable raw materials and ingredients |
|  |  |  | Energy density |
|  |  |  | Protein content |
|  |  |  | Carbohydrate content |
|  |  |  | Total fat content |
|  |  |  | Saturated fat content |
|  |  |  | Total sugar content |
|  |  |  | Added sugar content |
|  |  |  | Salt |
|  |  |  | Flavorants |
|  |  |  | Quality factors |
|  |  |  | Consistency and product size |
|  |  |  | Food additives |
|  |  |  | Contaminants |
|  |  |  |  |
|  |  |  | **Vitamins and Minerals including:** |
|  |  |  | Iron |
|  |  |  | Zinc |
|  |  |  | Copper |
|  |  |  | Selenium |
|  |  |  | Magnesium |
|  |  |  | Iodide |
|  |  |  | Calcium |
|  |  |  | Sodium |
|  |  |  | Vitamin A |
|  |  |  | Vitamin B 1 |
|  |  |  | Vitamin B 2 |
|  |  |  | Vitamin B 3 |
|  |  |  | Vitamin B 6 |
|  |  |  | Vitamin B 12 |
|  |  |  | Folic Acid |
|  |  |  | Vitamin C |
|  |  |  | Vitamin D |
|  |  |  | Vitamin E |
|  |  |  |  |
|  | 4 | Are there binding legal measures for the declaration of nutritional information on commercially produced foods intended for children 6 to 36 months of age? (This includes standards and legislation regarding the listing of the nutritive value of CPCF's on the nutrient information label and can include the energy value in kcal or kj, amount of protein, carbohydrate and fat, and the amount of vitamins and minerals contained in either numerical form per 100g or 100ml or as a percentage of the Recommended Dietary Intake. | |
|  |  | Yes | No |
|  |  |  |  |
|  | 5 | If no, are there binding legal measures for the declaration of nutritional information on commercially produced foods intended for the general population? | |
|  |  | Yes | No |
|  |  |  |  |
|  | 6 | If yes to either question 4 or 5, please include the binding legal measure for the following components: | |
|  |  | 6a. Declaration of nutritional information on the label of any processed foods | |
|  |  | Yes | No |
|  |  | 6b. Declaration of nutritional information on the label of commercially produced foods intended for children 6 to 36 months of age | |
|  |  | Yes | No |
|  |  |  |  |
|  |  | 6c. If yes to 6a or 6b: are there binding legal measures on the listing of nutrition information by 100g/100ml of the product or by % of RDI or other country standard for dietary intake (% DV, %RDA etc.) | |
|  |  |  |  |
|  | 7 | If yes to either 4 or 5, please include the binding legal measures for the following components: | |
|  |  |  | Recommended serving size |
|  |  |  | Ingredient’s list |
|  |  |  | Ingredients list in descending order of proportion (with the exception of vitamins and minerals which can be grouped together) |
|  |  |  | The percentage of the weight of each ingredient to the total (i.e., if the ingredients are apple, water and vitamins and minerals the list would be: apple (80%), water (20%) and vitamins and minerals (<0.01%). |
|  |  |  |  |
| Labelling | 8 | Are there binding legal measures for the labelling of commercially produced foods intended for children 6 to 36 months of age? | |
|  |  | Yes | No |
|  |  |  |  |
|  | 9 | If yes, please include the binding legal measures for the following components | |
|  |  |  | Minimum age of child intended for consumption of the product |
|  |  |  | Maximum age of child intended for consumption of the product |
|  |  |  | Use of images or text that suggests the product is suitable for babies under 6 months of age |
|  |  |  | Inclusion of a message on the importance of breastfeeding for up to two years or beyond |
|  |  |  | Use of images or text to suggest the product is equivalent or superior to breastmilk |
|  |  |  | Use of images or text to recommend the use or promotion of bottle feeding |
|  |  |  | For products with a spout, inclusion of a statement to the effect “infants and young children must not be allowed to suck from the container” |
|  |  |  | For products with a spout, statement that warns against choking hazard i.e., WARNING CHOKING HAZARD DO NOT LET BABIES AND CHILDREN PLAY WITH CAP |
|  |  |  |  |
|  | 10 | Are there binding legal measures for the use of nutrition/health or composition claims for foods intended for older infants and young children? | |
|  |  | Yes | No |
|  | 11 | If no to 10, Are there binding legal measures for the use of nutrition/health or composition claims for foods intended for the general population? | |
|  |  |  |  |
|  | 12 | If yes to 10 or 11, please provide including the binding legal measure for the following claims: | |
|  |  |  | Permitted compositional claims |
|  |  |  | Not permitted compositional claims |
|  |  |  | Nutrient content claims |
|  |  |  | Nutrient function claims |
|  |  |  | Disease risk reduction claims |
|  |  |  | Any other claims |
