# Supplementary material for: Overpromoted and underregulated: National binding legal measures related to commercially produced complementary foods in seven Southeast Asian countries are not fully aligned with available guidance
Source: Matern Child Nutr. 2023 Dec 13;19(Suppl 2):e13588. doi: 10.1111/mcn.13588 (PMC10719056; doi:10.1111/mcn.13588)
Supplement: Supplementary file 4 — Supporting Information. [file MCN-19-e13588-s003.docx]

**Supplementary Table 4** Comparison of nutrition composition content provided through Codex to the adapted NPM for CPCF product categories

|  | **No added sugar / sweetener** | **Low/no added fruit** | **Total sugar** | **Sodium standard** | **Energy density†** | **Protein standard** | **Total Fat** |
| --- | --- | --- | --- | --- | --- | --- | --- |
| Category 1 |  |  |  |  |  |  |  |
| 1.1 Dry or instant cereals/starch‡ | ‡ | ‡ | NA | ‡ | NA | ‡ | ‡ |
| Category 2 | | | | | | | |
| 2.1 Dairy-based desserts and cereal products |  |  | NA | § |  | NA |  |
| 2.2 Fruit purée with or without addition of vegetables, cereals, or milk |  |  | NA | § |  | NA |  |
| 2.3 Vegetable only purée |  |  | NA | § |  | NA |  |
| 2.4 Puréed vegetables and cereals |  |  | NA | § |  | NA |  |
| 2.5 Puréed meal with cheese (but not meat or fish) mentioned in the name |  |  | NA | § |  |  |  |
| 2.6 Puréed meal with meat or fish mentioned as first food in product name |  |  | NA | § |  |  |  |
| 2.7 Puréed meals with meat or fish (but not named as the first food in product name) |  |  | NA | § |  |  |  |
| 2.8 Purées with only meat, fish, or cheese in name of product |  |  | NA | § |  |  |  |
| Category 3 |  |  |  | § |  |  |  |
| 3.1 Meat, fish, or cheese-based meal with chunky pieces |  |  | NA | § |  |  |  |
| 3.2 Vegetable-based meal with chunky pieces |  |  | NA | § |  | NA |  |
| Category 4 |  |  |  | § |  |  |  |
| 4.1 Confectionery, sweet spreads and fruit chews ¶ | NA | NA | NA | NA | NA | NA | NA |
| 4.2 Fruit (fresh or dry whole fruit or pieces) |  | NA | NA | NA | NA | NA |  |
| 4.3 Other snacks and finger foods ‡ | ‡ | NA | ‡ | ‡ | NA | NA | ‡ |
| Category 5 ¶ |  |  |  |  |  |  |  |
| 5.1 Single or mixed fruit juices, vegetable juices, or other non-formula drinks | NA | NA | NA | NA | NA | NA | NA |
| 5.2 Cow’s milk and milk alternatives with added sugar or sweetening agent | NA | NA | NA | NA | NA | NA | NA |

No binding legal measures Partial alignment Full alignment

† Energy density guidelines are provided in “Guidelines on formulated complementary foods for older infants and young children, adopted in 1991, CAC/GL 8-1991. Amended in 2017. Revised in 2013.” However, they exclude CPCF previously defined under CXS 74-1981 and CXS 73-1981.

‡ Standard for processed cereal-based foods for infants and young children, adopted in 1981, CXS 74-1981. Revised in 2006. Amended in 2017, 2019.

§ Standard for canned baby foods, adopted in 1981, CXS 73-1981. Amended in 1983, 1985, 1987, 1989, 2017.

¶ Categories 4.1, 5.1 and 5.2 do not have nutrition composition requirements in the adapted NPM for CPCF food categories as they are prohibited from sale.

NA stands for not applicable as no thresholds or recommendations exist in the adapted NPM for CPCF food categories.
