# Supplementary material for: Overpromoted and underregulated: National binding legal measures related to commercially produced complementary foods in seven Southeast Asian countries are not fully aligned with available guidance
Source: Matern Child Nutr. 2023 Dec 13;19(Suppl 2):e13588. doi: 10.1111/mcn.13588 (PMC10719056; doi:10.1111/mcn.13588)
Supplement: Supplementary file 5 — Supporting Information. [file MCN-19-e13588-s001.docx]

**Supplemental Table 5** Comparison of labelling practices recommendations based on Codex standards/guidelines and the WHO Guidance to the adapted CPCF NPM by product category

| Labelling recommendations | Dry or instant cereals/ starch | Soft–wet spoonable,  ready-to-eat foods | Meals with chunky pieces | Dry finger foods and snacks |
| --- | --- | --- | --- | --- |
| *Protection and promotion of breastfeeding* | | | | |
| Has a minimum recommended age of introduction of at least 6 months | † | † | † | † |
| Not marketed as suitable for <6 months | † | † | † | † |
| Message on importance of breastfeeding ≥2 years2 years | † | † | † | † |
| Does not suggest superiority or equivalence to breastmilk | † | † | † | † |
| Does not recommend or promote bottle feeding | † | † | † | † |
| *Claims* | | | | |
| No non-permitted compositional claims | ‡ | ‡ | ‡ | ‡ |
| No nutrient content claims | ‡ | ‡ | ‡ | ‡ |
| No nutrient function claims | ‡ | ‡ | ‡ | ‡ |
| No disease risk reduction claims | ‡ | ‡ | ‡ | ‡ |
| No other claims | ‡ | ‡ | ‡ | ‡ |
| *Product name and ingredient list* | | | | |
| Product name reflects ingredients in descending order as per ingredient list | § | ¶ | ¶ | § |
| Percentage of fruit stated in ingredient list | # | # | # | # |
| Percentage of added water stated in ingredient list | # | # | # | # |
| Percentage of protein stated in ingredient list | # | ¶, # | # | # |
| *Messages on products with a spout* | | | | |
| Product with spout states not to suck from the container |  |  |  |  |
| Product with spout warns that cap is a choking hazard |  |  |  |  |
| *Age restriction on blended/puréed products* | | | | |
| Maximum recommended age of use of 12 months |  |  |  |  |

No binding legal measures Partial alignment Full alignment

† WHO. (2016). Maternal, infant and young child nutrition: Guidance on ending the inappropriate promotion of foods for infants and young children. Geneva: World Health Organization.

‡ Codex Alimentarius Commission. (1997). Guidelines for use of nutrition and health claims. CAC/GL 1997. Revised in 2004, 2008, 2009, 2010, 2011, 2012, and 2013.

§ Codex standard for processed cereal-based foods for infants and young children, adopted in 1981, CXS 74-1981. Revised in 2006. Amended in 2017, 2019.

¶ Codex Alimentarius Commission. (1981). Codex Standard for canned baby foods. Codex STAN CSX 73-1981. Amended in 1983, 1985, 1989, 2017.

# Codex Alimentarius Commission. (2018). General standard for the labelling of prepackaged foods. CXS 1-1985. Amended in 1991, 1999, 2003, 2005, 2008, 2010. Revised in 2018.
